# Rituximab mechanisms of action in B-CLL: a new piece of the puzzle

**DOI:** 10.18632/oncotarget.26016

**Published:** 2018-08-28

**Authors:** Hervé Watier

**Affiliations:** Hervé Watier: Université de Tours, Tours, France

**Keywords:** rituximab, B-CLL, pharmacodynamics, complement, resistance

More than 20 years after its approval, determinants of rituximab (RTX) pharmacodynamics remain poorly known, and the quest to find them seems to be an endless story. Such an understanding is crucial though, both for guiding the development of new anti-CD20 antibodies or of new combinations and for personalising treatments. In this issue of *Oncotarget*, Anne Bordron and colleagues provide a new and rather unexpected information. Indeed, they show that expression of α6-sialylated *N*-glycans at the surface of malignant B cells in chronic lymphocytic leukaemia (B-CLL) is associated with resistance to complement-dependent cytotoxicity (CDC) in the presence of RTX (RTX-CDC), and that this resistance is associated with a lower clinical response to RTX plus chemotherapy.

The clinical response was analysed in a monocentric cohort of 34 patients, whose B-CLL cells were evaluated for RTX-CDC before treatment. Despite the limited number of patients, they show that, in comparison with patients of the RTX-CDC-normal group (*n* = 23), patients of the RTX-CDC-resistant group (*n* = 11) have a lower complete response rate (65.2%, *versus* 100%, *p* = 0.03), and a lower time to relapse (median TTR: 45 months, *vs* >80, *p* = 0.03). Complement activation very likely contributes to the response to RTX, since the clinical response of RTX-CDC-resistant B-CLL patients to RTX and chemotherapy does not seem to depart from the one of patients treated with chemotherapy. It is therefore the first time that RTX-CDC is associated with the clinical response to RTX in B-CLL patients. However, nobody knows yet whether complement activation occurs *in vivo* throughout the treatment period or only at specific moments, particularly when the circulating RTX concentrations are high, or whether complement acts *in vivo* mostly through C3b/C3bi opsonisation or formation of the membrane attack complex. More importantly, the results does not exclude the crucial contribution of other mechanisms of action, such as direct apoptosis or FcγR-dependent mechanisms. The latter are boosted in obinutuzumab at the expense of its capacity to activate complement, without any loss of efficacy in B-CLL, in comparison with RTX [1]. On the contrary, CDC activity is enhanced in ofatumumab at the expense of direct apoptosis, and this monoclonal antibody displays activity in B-CLL. It is trite to say that multiple mechanisms of action contribute to the efficacy of anti-CD20 monoclonal antibodies; however, this is the first convincing demonstration of complement contribution to RTX activity in B-CLL.

What is particularly interesting in this article is that the experimental conditions implemented to evaluate RTX-CDC were very carefully standardised. They managed to hedge against defaults such as underdosing (use of 10µg/mL RTX), complement deficiency (use of 20% frozen AB serum from a pool of donors) or insufficient incubation time (24hr). In other terms, only the intrinsic resistance - or not - of patient B-CLL to RTX-CDC was evaluated with this assay. The read-out (cell lysis) was shown to be dependent on the formation of complement membrane attack complex since it is abrogated by eculizumab, an anti-C5 monoclonal antibody. However, it is less clear whether this CDC only depends on the classical pathway (C4b2a3b as a C5 convertase) or whether it also involves the amplification loop, which is based on proteins of the alternative pathway (C3bBb3b as an additional C5 convertase). However, such an assay is feasible in many labs, and could be implemented for future multicentric studies, in order to reproduce these data on a larger scale while working on freshly isolated cells.

Anne Bordron and colleagues systematically analysed every factor known or reputed to be associated with complement activation by anti-CD20 antibodies: expression of complement regulatory proteins, markers of lipid rafts, and sialic acid expression on B-CLL cells. Only the latter was found to be associated with resistance to RTX-CDC in the 69 analysed samples. Once again, this is the first time such an association is found, but it is not the only surprising information. Admittedly, loss of sialic acids is known to be associated with an increased susceptibility to CDC for a long time [2, 3] and more recent findings have shed light on the recognition of membrane sialic acids bind factor H, a soluble protein that regulates complement at the level of C3b [4, 5]. However, factor H binds sialic acids in α3-linkage [4, 5], those that are recognised by the lectin of *Maackia amurensis* and that are not shown presently to be associated with resistance of B-CLL cells to RTX-CDC! Moreover, recruitment of factor H does differ between samples susceptible or resistant to RTX-CDC. In reality, Anne Bordron and colleagues show that B-CLL resistance to RTX-CDC is associated with the expression of α6-linked sialic acids present in *N*-linked glycans on membrane glycoproteins, which are detected by the *Sambucus nigra* lectin (*p* = 0.005). Moreover, there is a very limited overlap of *Sambucus nigra* agglutinin binding levels between RTX-CDC-susceptible or resistant samples (Figure 4A). No obvious explanation can be currently provided explaining how α6-linked sialic acids in *N*-glycans down-regulate complement activation. However, detecting RTX-CDC-resistant B-CLL by simply using a biotinylated or even a fluorescent lectin is nothing other than very simple, and could be easily introduced in clinical trials, to confirm the data and, if confirmed, to help at stratifying patients and at personalising treatments.

The Brest group also clearly shows that the α6-sialyltransferase enzymatic activity is increased in RTX-CDC-resistant B-CLL. Such an enzymatic activity is dependent on *ST6GAL1* or *ST6GAL2*, the 2 genes coding for a α6-sialyltransferase. Currently, nobody knows which one is overexpressed in CDC-resistant B-CLL cells (nor in any B cell subpopulation), and whether this overexpression could be genetically determined or not. Interestingly, *ST6GAL1* polymorphisms have already been associated with IgG glycosylation [6] and IgA nephropathy [7], suggesting that this gene could be differentially regulated within the B cell lineage. BCR *N-*glycosylation is altered in *IGHV*-unmutated B-CLL cells [8], with an increased expression of highly-mannosylated *N*-glycans, but this does not appear to be linked with the current observation since RTX-CDC resistant B-CLL cells are more frequently unmutated, an observation confirmed by Anne Bordron and colleagues, and because highly-mannosylated *N*-glycans cannot be sialylated (sialylation only occurs in complex-type *N*-glycans). Many more membrane proteins other than the BCR could be α6-sialylated, and could explain the observation. However, like other ones, α6-sialylated BCR could be the ligand in cis (expressed on the same cell) of a particular Siglec, CD22, an inhibitory receptor that recognises the α6-sialylated *N*-glycans of adjacent proteins such as CD22 itself, CD45 or BCR [9]. Whether CD22 expression plays a role in protecting B-CLL cells from RTX-CDC remains to be determined. CD22 is weakly expressed on B-CLL cells and has never drawn particular attention, but it tends to be increased in the unmutated subgroup [10].

In conclusion, this interesting article combines a new clinical finding with the description of a new biomarker of resistance, and broadens our perspectives concerning the membrane environment and proteins dynamics that could influence the response to RTX.
